# Acidic and hypoxic tumor microenvironment regulation by CaO_2_-loaded polydopamine nanoparticles

**DOI:** 10.1186/s12951-022-01752-8

**Published:** 2022-12-28

**Authors:** Shuangrong Ruan, Weimin Yin, Jiao Chang, Yan Yang, Jiuyuan Sun, Xiaoyi Ma, Ying Liu, Jie Zang, Yiqiong Liu, Yongyong Li, Tianbin Ren, Haiqing Dong

**Affiliations:** 1 Key Laboratory of Spine and Spinal Cord Injury Repair and Regeneration, Ministry of Education, School of Medicine, Tongji Hospital, The Institute for Biomedical Engineering & Nano Science, Tongji University, 389 Xincun Road, Shanghai, 200092 China; 2 Shanghai Skin Disease Hospital, School of Medicine, Tongji University, Shanghai, 200092 China

**Keywords:** Lactic acid, Hypoxia, Mesoporous polydopamine, Antitumor metastasis

## Abstract

Hypoxia and high accumulation of lactic acid in the tumor microenvironment provide fertile soil for tumor development, maintenance and metastasis. Herein, we developed a calcium peroxide (CaO_2_)-loaded nanostructure that can play a role of “one stone kill two birds”, i.e., acidic and hypoxic tumor microenvironment can be simultaneously regulated by CaO_2_ loaded nanostructure. Specifically, CaO_2_-loaded mesoporous polydopamine nanoparticles modified with sodium hyaluronate (denoted as CaO_2_@mPDA-SH) can gradually accumulate in a tumor site. CaO_2_ exposed in acidic microenvironment can succeed in consuming the lactic acid with oxygen generation simultaneously, which could remodel the acid and hypoxia tumor microenvironment. More importantly, the relief of hypoxia could further reduce lactate production from the source by down-regulating the hypoxia inducible factor-1α (HIF-1α), which further down-regulated the glycolysis associated enzymes including glycolysis-related glucose transporter 1 (GLUT1) and lactate dehydrogenase A (LDHA). As a result, CaO_2_@mPDA-SH alone without the employment of other therapeutics can dually regulate the tumor hypoxia and lactic acid metabolism, which efficiently represses tumor progression in promoting immune activation, antitumor metastasis, and anti-angiogenesis.

**Supplementary Information:**

The online version contains supplementary material available at 10.1186/s12951-022-01752-8.

## Introduction

Lactic acid, aerobic glycolysis product in the tumor microenvironment (TME) was once considered as a waste, plays an essential role in tumors survival, growth, and metastasis [1–3]. On the one hand, lactic acid can provide fuel for the oxidative metabolism of oxidized tumor cells [4, 5], and up-regulate vascular endothelial growth factor (VEGF) in tumor cells and IL-8 in endothelial cells through a series of signaling pathways [6], synergically acting on lactic acid-induced tumor angiogenesis [7], thus facilitating tumor invasion and metastasis [8, 9]. On the other hand, lactic acid also plays immunosuppressive role in the tumor [10]. The lactic acid metabolized by tumor cells promotes the acidification of the tumor microenvironment [11], directly affects the signaling pathway of immune cells, and ultimately leads to inhibition in activation and proliferation of various immune cells, which subsequently causes immune evasion [12–14]. Therefore, targeting lactate metabolism is recognized as a promising tumor therapeutic strategy. Lactate oxidase (LOX), an enzyme utilized to directly deplete the lactic acid in the tumor stroma, however, requires to consume oxygen when oxidizing lactic acid [15], which will aggravate the hypoxia of solid tumors.

Hypoxia is known to upregulate expression of hypoxia-inducible factor 1α (HIF-1α), a transcription factor that can up- and down-regulate many genes involved in cancer metabolism, and enhance the expression of numerous tumor markers, co-regulate cancer cell proliferation, metastasis, and invasion [16–19]. Previous studies demonstrated that suppressing the accumulation of HIF1-α in breast cancer cells via gene knockout method can inhibit the expression of GLUT1 and LDHA [20]. GLUT1 increases glucose uptake by tumor cells and produces more lactic acid via glycolysis, whereas LDHA favors the conversion of pyruvate into lactate [21, 22], the presence of GLUT1 and LDHA can enhance lactic acid production at the source. Consequently, it is quite difficult to achieve an anti-tumor effect if only depleting lactic acid without improving hypoxia or in combination with other therapies. To resolve this contradiction, Tang et al. combined LOX with the hypoxic sensitive prodrug AQ4N. Lactic acid consumption catalyzed by LOX resulted in hypoxia which can activate AQ4N to its anticancer form of AQ4 for enhanced chemotherapy [23]. Additionally, more researchers utilize the dismutation of endogenous hydrogen peroxide (H_2_O_2_) to produce oxygen, thus ameliorating the tumor hypoxia [24, 25].

**Scheme 1:**
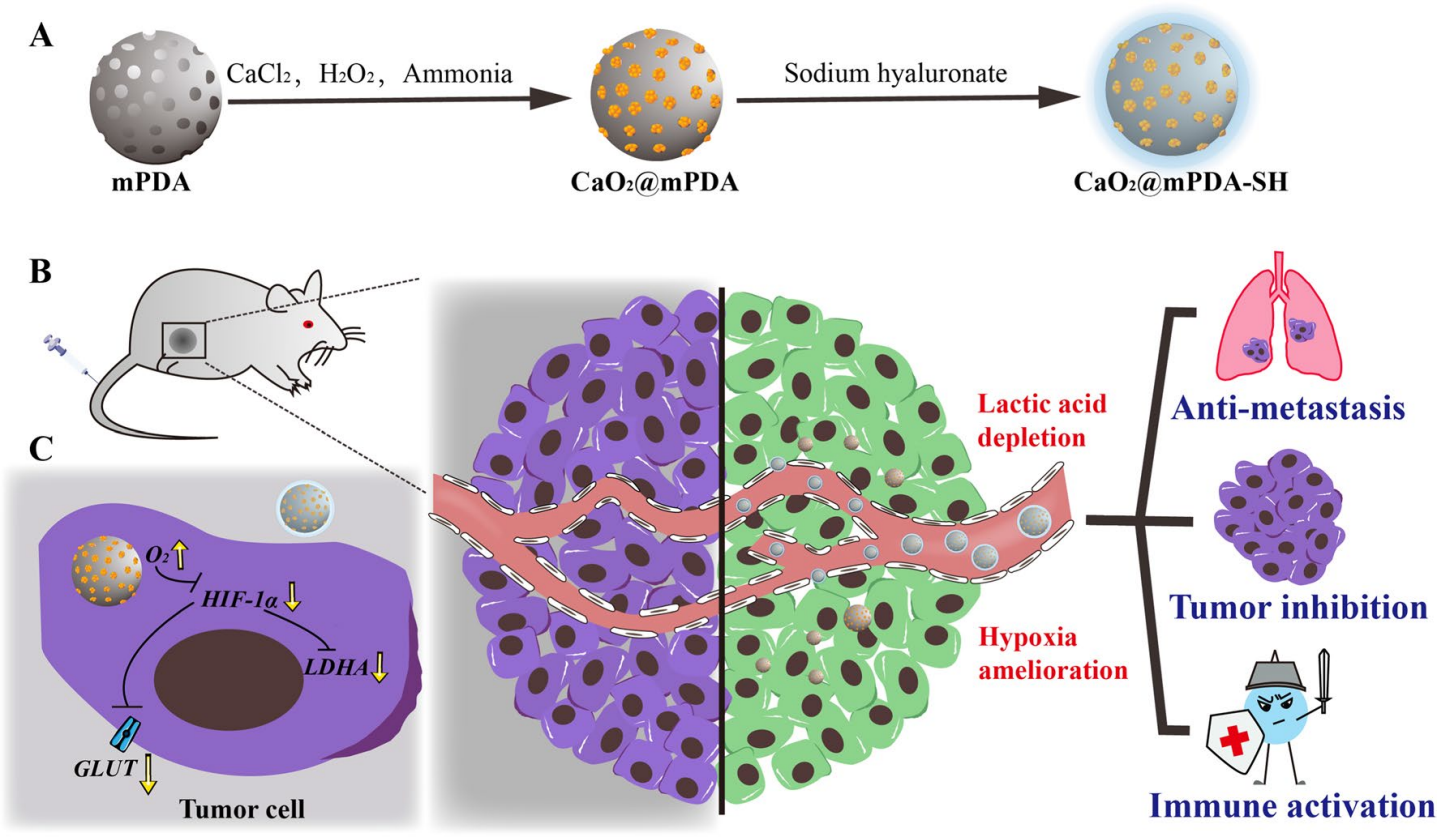
Schematic illustration of CaO_2_@mPDA-SH nanoparticles dually regulate acidic and hypoxic tumor microenvironments. **A** Preparation steps of CaO_2_@mPDA-SH nanoparticles. **B** CaO_2_@mPDA-SH nanoparticles accumulate in tumor sites via the EPR effect, CaO_2_ exposed in acidic microenvironment can consume acid with generating oxygen, thereby allowing for the achievement of tumor growth inhibition, immune activation and antitumor metastasis effect. **C** The relief of hypoxia can further reduce lactic acid production at the source by down-regulating the expression of HIF-1α, which further down-regulated the expression of glycolysis associated enzymes including GLUT1 and LDHA

Calcium peroxide (CaO_2_), capable of generating oxygen or H_2_O_2_ with Ca^2+^ release, has been widely used in tissue engineering, antibacterial and antitumor therapy [26], such as (1) supplying oxygen for photodynamic therapy; (2) enhancing the efficacy in combination with chemotherapeutic agents (e.g., DOX); (3) mediating chemodynamic therapy as a solid H_2_O_2_ supplier; (4) calcification with other materials [27]. In this study, based upon properties of CaO_2_, we proposed a novel strategy of consuming lactic acid accompanied by the production of oxygen to improve tumor acidic and hypoxic microenvironment simultaneously. As shown in Scheme 1A, mesoporous polydopamine (mPDA) nanoparticles with good biocompatibility and outstanding wet adhesion properties were harnessed to load CaO_2_. To protect CaO_2_ from premature decomposition or leakage, the obtained nanostructure (denoted as CaO_2_@mPDA) was then coated with sodium hyaluronate (SH) to form the novel potential nanoparticles (named as CaO_2_@mPDA-SH). After intravenous injection (i.v.) into 4T1 tumor-bearing mice (Scheme 1B), nanoparticles could extravasate into tumor tissues efficiently via the enhanced permeability and retention (EPR) effect. CaO_2_ was exposed to acidic milieu and reacted with lactic acid with oxygen generating, thereby allowing for the achievement of tumor growth inhibition, immune activation and antitumor metastasis effect. In addition, the relief of hypoxia could further reduce lactic acid production at the source by down-regulating the expression of HIF-1α, which further down-regulated the expression of glycolysis associated enzymes includes GLUT1 and LDHA (Scheme 1C). Such CaO_2_@mPDA-SH nanoparticles were expected to efficiently regulate the tumor lactic acid metabolism to repress tumor progression without the employment of other therapies.

## Materials and methods

### Materials

Triblock poly (ethylene oxide)-bpoly(propylene oxide)-b-poly(ethylene oxide) Pluronic F127 (EO_106_PO_70_EO_106_, Mav = 12,600) and dopamine hydrochloride (C_8_H_11_NO_2_·HCl, DA·HCl) were brought from Sigma-Aldrich. Ethanol (C_2_H_6_O) was purchased from Sinopharm Chemical Reagent Co., Ltd. 1,3,5-trimethylbenzene (C_9_H_12_), Calcium chloride, hydrogen peroxide (H_2_O_2,_ 30 wt% solution in water), ammonia water (25–28%), *N*-2-hydroxyethyl piperazine-*N*′-2-ethanesulfonic acid (HEPES), Hyaluronic acid sodium salt from Streptococcus equi, Rhodamine B (RhB) and dichloride [Ru(dpp)_3_]Cl_2_ were purchased from Aladdin (Shanghai, China). The lactate assay kit was brought from Nanjing Jiancheng Bioengineering Institute. Cell Counting Kit-8 (CCK-8), the Annexin V-FITC Apoptosis Detection Kit, and the Calcium Colorimetric Assay Kit were obtained from Beyotime Biotechnology Co., Ltd. Anti-CD16/32, anti-CD45-PE, anti-CD11b-APC, anti-CD11c-APC anti-F4/80-PE/Cy7, anti-CD80-FITC, anti-CD206-PE, anti-CD86-PE, anti-MHCII-V450, anti-CD3-APC, anti-CD4-FITC, and anti-CD8-PE/Cy7 were purchased from BioLegend. ELISA kits were purchased from Dakewei Biotech Co., Ltd.

All chemicals were of analytical grade and used without further purification. Deionized water (18.2 MΩ·cm resistivity at 25 °C) was used for all experiments.

### Cells and animals

The murine 4T1 breast cancer cells were cultured in DMEM containing 10% FBS and 1% penicillin–streptomycin and were incubated at 37 °C under a humidified atmosphere containing 5% CO_2_.

BALB/c mice (female, 5–6 weeks old) were ordered from the Shanghai Laboratory Animal Center (SLAC, Shanghai, China) and bred in a sterile, specific pathogen-free (SPF) laboratory at Tongji University. The experimental research was carried out in accordance with relevant guidelines and regulations and all animal procedures in compliance with the guidelines of the Institutional Animal Care and Use Committee of Tongji University.

### Synthesis of mPDA

The synthesis of mPDA was carried out according to our previous work [28], firstly, 500 mg DA and 1000 mg Pluronic F127 were dissolved in 100 mL 50% ethanol under stirring. Then, 2 mL of TMB was added drop by drop into the solution. After 30 min of stirring, 5 mL of ammonia solution was introduced slowly with continuous stirring for 2 h at room temperature. Then the products were purified by centrifugation, washed at least three times with 50% ethanol to remove the template F127. The final separated products were suspended in water for further use.

### Synthesis and characterization of CaO_2_@mPDA-SH, Ca(OH)_2_@mPDA-SH and CaO_2_@mPDA

0.2 mg mPDA was added in a glass vial, then 500 μL 0.5 mM CaCl_2_ aqueous solutionand 125 μL 0.5 mg/mL sodium hyaluronate solution were added during stirring, later 100 μL 28–30% ammonia was added and stirred, 360 μL 30% H_2_O_2_ was finally added dropwise for 30 min. The reaction mixture was subjected to centrifuge at 13,000 rpm for 15 minto discard the supernatant, the residual was then resuspended with HEPES buffer (20 mM HEPES, 150 mM NaCl, pH 7.4) and washed twice, thus obtained CaO_2_@mPDA-SH dispersion in HEPES buffer. To obtain unmodified CaO_2_@mPDA NPs, the sodium hyaluronate solution was just replaced by deionized water in the above procedure. And the preparation of Ca(OH)_2_@mPDA-SH was carried out according to the above steps, without additional injection of hydrogen peroxide.

The morphology of the formed CaO_2_@mPDA-SH, mPDA was characterized by a transmission electron microscope (TEM, JEM-1230). The size and zeta potential were determined by dynamic light scattering (DLS, ZS90, Malvern) at 25 °C.

### Stability

The stability of CaO_2_@mPDA-SH was evaluated by monitoring the size distribution in HEPES buffer for 7 days. Size variations were measured by DLS at 2, 4, 8, 12, 24 hour 2, 3, 4, 5, 6, 7 days, respectively.

### Hemolysis evaluation of CaO_2_@ mPDA-SH

The hemolysis ratio of CaO_2_@ mPDA-SH was estimated on the 5% red blood cells (RBCs). Nanoparticles were dispersed with PBS (pH 7.4) into CaO_2_@mPDA-SH solutions (500 μL) of concentration from 25 μg mL^−1^ to 1000 μg mL^−1^, and then mixed with RBC suspension (500 μL). PBS (500 μL) and water (500 μL) mixed with equal volume of RBC suspension to act as the negative and positive controls, respectively. The above samples were kept at 37 °C for 3 h with subsequent centrifugation (3000 rpm, 1 min). The absorbance of the supernatants in different groups was measured at 540 nm by UV–visible spectroscopy (Varian, Ltd., Hong Kong). And hemolysis ratio was calculated according to the following equation (A denotes the absorbance of different groups):

$${\text{Hemolysis}}\;{\text{ratio}}\;(\% ) \, = \frac{{{\text{A}}^{{{\text{sample}}}} - {\text{A}}^{{{\text{negative}}}} }}{{{\text{A}}^{{{\text{positive}}}} - {\text{A}}^{{{\text{negative}}}} }} \times 100\%$$

### In vitro cytotoxicity of CaO_2_@mPDA-SH

The murine 4T1 breast cancer cells were inoculated on 96-well plates (1 × 10^4^ cells per well) with cell culture medium DMEM for 24 h. Afterward, the DMEM was replaced by samples (CaO_2_@mPDA-SH, mPDA) dispersion in DMEM with different concentration gradients (0, 10, 20, 50, 100, 200, and 400 μg mL^−1^, respectively), after 24 h incubation, the medium was discarded and CCK-8 test was performed to assess the cell viability.

### Cellular uptake

Firstly, mPDA nanoparticles were labeled with RhB. Specifically, mPDA (3.0 mg) and RhB with mass ratio is of 10:1 were mixed in sodium bicarbonate solution (0.1 M) and stirred at 300 rpm in the dark. After 12 h, the RhB-labeled nanoparticles were centrifuged (13,500 rpm, 15 min) and washed with deionized water for at least three times to obtain RhB-mPDA. RhB-labeled CaO_2_@mPDA-SH nanosystems were also obtained via the above similar method. For the evaluation of cellular uptake, 4T1 cells were seeded in 24-well microplates (1 × 10^5^ cells per well). After 24 h incubation, the cells were incubated with fresh medium composed of RhB-mPDA (100 µg mL^−1^) for 1, 2, 4, 5, and 7 h, respectively. In addition, cytochalasin D (10 × 10^–6^ M) was used for investigating the cell phagocytosis. After washing with PBS for 3 times, the RhB fluorescence was determined by flow cytometry (FACSVerse, BD).

### Cell apoptosis assay

4T1 cells were incubated in a 6-well plate (1 × 10^6^ cells well^−1^) for 24 h and treated with samples (CaO_2_@mPDA-SH, mPDA) of different concentration gradients (0, 10, 20, 50, 100, 200, and 400 μg mL^−1^, respectively). After 12 h cultivation, the culture medium was swilled with PBS several times and cells were digested by trypsin without EDTA. Cells were then subjected to flow cytometry to detect apoptosis cells stained with annexin V-FITC/PI.

### In vitro cellular uptake of CaO_2_@mPDA-SH

The cellular uptake behavior of CaO_2_@mPDA-SH in 4T1 cells was quantitatively analyzed by flow cytometry (Guava easyCyte) and visualized by fluorescent microscopy (Lionheart FX automated live cell imager, BioTek). Briefly, the 4T1 cells were seeded in a 24-well plate (2 × 10^5^ cells·well^−1^) for 24 h and incubated with different samples (CaO_2_@mPDA-SH and mPDA) for 1, 2, and 4 h. The 4T1 cells were collected and quantified by flow cytometry. Besides, the cells incubated with the CaO_2_@mPDA-SH were fixed with 4% paraformaldehyde solution and stained with DAPI for fluorescence microscope observation.

### In vitro lactic acid reduction

4T1 cells were seeded into the 48-well plate (2 × 10^4^ cells·well^−1^) for 24 h. Then, the cell supernatant was displaced with the fresh culture medium containing different formulations of mPDA, CaO_2_@mPDA, Ca(OH)_2_@mPDA-SH, and CaO_2_@mPDA-SH nanoparticles. After 4 h, cell supernatant samples of cells with different treatments were collected, and lactate content was detected using the Lactic Acid Assay Kit.

### In vitro hypoxia improvement

1 × 10^6^ 4T1 cells were seeded in a 6-well plate and incubated overnight and then treated with mPDA or CaO_2_@mPDA-SH for 4 h, respectively. After different treatments, cells were stained with DAPI and [Ru(dpp)_3_]Cl_2_ for visualizing the nuclei and hypoxia under a fluorescence microscope.

### In vivo imaging

The tumor-bearing mice were injected with Ce6-labeled nanoparticles (CaO_2_@mPDA-SH-Ce6) via the tail vein (5 mg/kg). The biodistribution of nanoparticles was monitored using an IVIS imaging system (Caliper PerkinElmer, Hopkinton, USA) at the predetermined time points. At the experiment endpoint, the mice were sacrificed, and the tumor and organs were harvested for ex vivo imaging. The pharmacokinetics study was also executed. CaO_2_@mPDA-SH-Ce6 were injected by tail vein injection with the same dose for each mice. The mice were anesthetized and 0.1 mL of blood was collected by retro-orbital bleeding at predetermined time points. The plasma was obtained by centrifugation (5000 rpm, 10 min) and the Ce6 concentration was measured by a fluorescence spectrophotometer (F-4600, HITA-CHI, Japan).

### In vivo antitumor efficacy and immune response evaluation

The orthotopic breast cancer mouse model was established first. 4T1 cells (2 × 10^6^ cells per mouse) were implanted into the breast region of female BALB/c mice. When the tumor volume reached 50 mm^3^, tumor-bearing mice were randomly divided into 5 groups (5 mice in each group). In the following 15-day observation period, each group was i.v. injection one of the followings of 100 µL on the 1st, 3rd, 5th day, respectively: (i) saline; (ii) mPDA (50 mg/kg, dispersed in HEPES buffer); (iii) CaO_2_@mPDA NPs (50 mg/kg, dispersed in HEPES buffer); (iv) Ca(OH)_2_@mPDA-SH NPs (50 mg/kg, dispersed in HEPES buffer); (v) CaO_2_@mPDA-SH NPs (50 mg/kg, dispersed in HEPES buffer). Body weight and tumor volume were measured every 2 days. And the tumor volume was calculated according to the equation of V = W·S^2^/2 (where W and S represented the longest diameter and the shortest diameter, respectively). Tumor inhibition ratio was defined as (V_PBS_ − V_T_)/V_PBS_ (where V_t_ represented the final tumor volume of other treatment group.

At the end of the treatment, all mice were sacrificed. Tumors, draining lymph nodes (DLNs) and spleen were collected for flow cytometry and immunofluorescence staining. Tumor tissue was mainly used to analyze the expression level of T cell infiltration (CD3^+^CD8^+^) and tumor-associated macrophage (M1 and M2), while the lymph node was mainly utilized to analyze DC maturation and spleen was used to analyze the relative abundance of CD8^+^ and CD4^+^ T cell subsets. Simultaneously, the main organs (heart, liver, spleen, lung and kidney) were collected for hematoxylin and eosin (H&E) analysis.

### Intratumor lactic acid content test

100 mg of tumor samples, collected 15 days after treatment, were homogenized in PBS. Following the centrifugation of tissue homogenates, the supernatant of tumor tissues was collected and detected via the Lactic Acid assay Kit.

### In vivo anti-metastasis and anti-angiogenesis efficacy

4T1 cells (2 × 10^6^ cells per mouse) were implanted into the breast region of female BALB/c mice. When the tumor volume reached 50 mm^3^, tumor-bearing mice were randomly divided into the same 5 groups (5 mice in each group), each group was administered nanoparticles intravenously on the 1st, 4th, 7th, 11th day, respectively. The dosages were consistent with the above. After the therapy for 28 days, all the mice suffered mercy killing. The tumors were harvested, Paraffin-embedded, deparaffinized and hydrated followed by antigen retrieval for immunohistochemistry staining against CD31 and VEGF. The lung was also harvested, fixed in 4% paraformaldehyde, sectioned, and stained with H&E for evaluating the anti-metastasis efficacy.

### Statistical analysis

All data in the present study were presented as mean ± S.D. All animal studies were performed after randomization. Using unpaired student’s t-test to appraise statistically significant discrepancies between two groups. One-way analysis of variance with Bonferroni tests for multiple group comparison. Significant differences were indicated by *P < 0.05, **P < 0.01, and ***P < 0.001.

## Results and discussion

### Characterization of CaO_2_@mPDA-SH

**Fig. 1:**
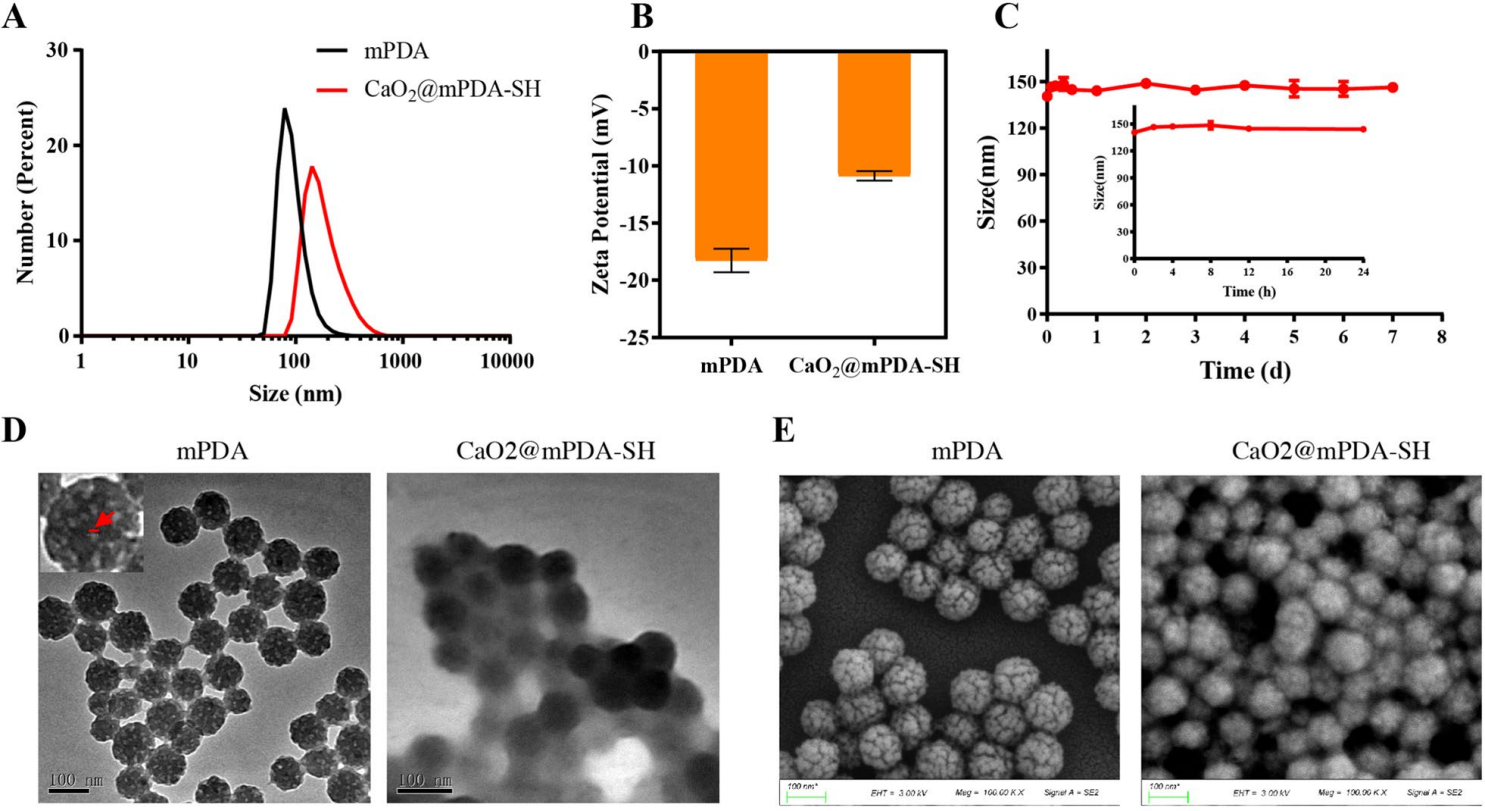
Characterization of CaO_2_@mPDA-SH nanoparticles. **A** Number-average hydrodynamic diameter distribution of mPDA and CaO_2_@mPDA-SH nanoparticles. **B** ζ potential of mPDA and CaO_2_@mPDA-SH nanoparticles dispersed in aqueous solution. **C** Stability of CaO_2_@mPDA-SH in HEPES buffer. **D** TEM images (insert is one magnified NP with pore size indicated in red and calculated by ImageJ) and **E** SEM images of mPDA and CaO_2_@mPDA-SH nanoparticles

The fabrication process of CaO_2_@mPDA-SH was illustrated in Scheme 1A. CaO_2_@mPDA were obtained via a one-step synthesis by successively adding ammonia and hydrogen peroxide (H_2_O_2_) dropwise in mPDA dispersion with calcium chloride (CaCl_2_) with. During the reaction, the color of the solution gradually changed from black to yellowish-brown, indicating the successful CaO_2_ loading (Additional file 1: Fig. S1). Different feeding mass ratios of mPDA and CaCl_2_ were used to screen the best feeding ratio of 1:50 with higher CaO_2_ content (Additional file 1: Fig. S1), To prevent premature leakage of CaO_2_ in the blood vessels, sodium hyaluronate was modified on the surface of CaO_2_@mPDA NPs via physical absorption such as hydrogen bonding and electrostatic interaction as a protective layer (named CaO_2_@mPDA-SH). The FTIR characteristic absorption spectrum peaks around 880 cm^−1^ indicated the existence of peroxide groups (Additional file 1: Fig. S4). Moreover, the X-ray diffraction (XRD) characterization displayed that the diffraction peaks of the CaO_2_@mPDA NPs are in accord with those of the CaO_2_ standard card (PDF#01-085-0514), which demonstrated the successful formation of CaO_2_ (Additional file 1: Fig. S5). And the loading rate of CaO_2_ was calculated to be 40.92% via the calcium colorimetric assay kit. The dynamic light scatter (DLS) and ζ-potential were used to characterize the nanoparticles (NPs) with a uniform hydrodynamic diameter of 147 nm and ζ potential of − 10.8 mV (Fig. 1A, B). To examine the stability of CaO_2_@mPDA-SH in solution, we monitored the size changes of nanoparticles in HEPES buffer stored at 4 °C. The results showed that the mean particle size of CaO_2_@mPDA-SH was almost unaltered throughout 7 days monitoring (Fig. 1C). To investigate the morphology of synthesized NPs, TEM pictures (Fig. 1D) showed the mesoporous structure of mPDA with mean diameter about 93 nm, a close examination of surface suggests that pores with mean size around 11 nm, yet the non-porous structure of CaO_2_@mPDA-SH (~ 95 nm) could be visibly observed, which mainly due to the existence of sodium hyaluronate layer. Furthermore, the size from SEM images is consistent well with the morphology of nanoparticles (Fig. 1E). Hemolytic toxicity tests showed that while the concentration of CaO_2_@mPDA-SH was below 200 μg/mL, the hemolysis rate was lower than 5% (Additional file 1: Figs. S2, S3), indicating that CaO_2_@mPDA-SH has definite biosafety through intravenous administration below the concentration range.

### In vitro cytotoxicity of CaO_2_@mPDA-SH

**Fig. 2:**
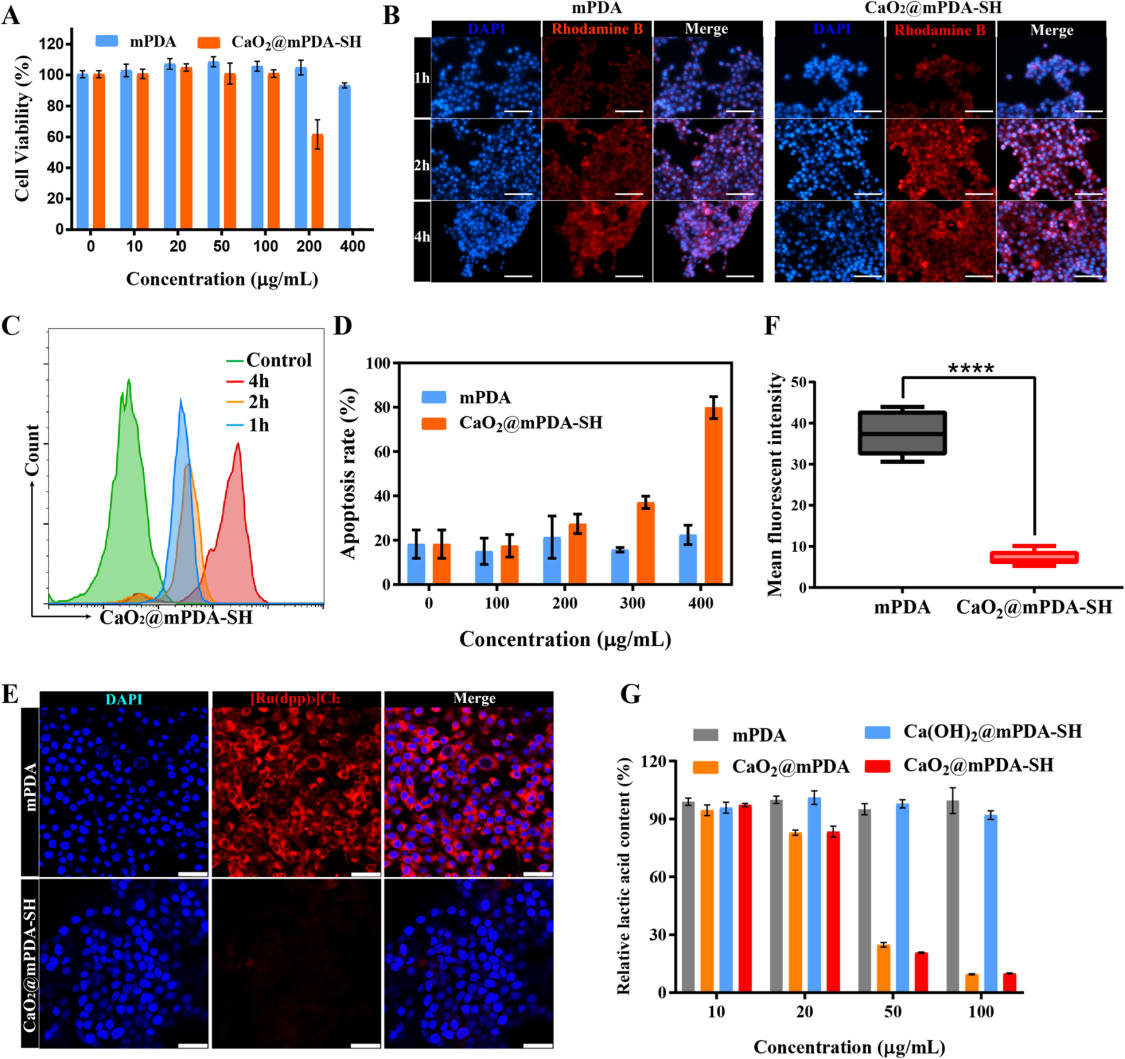
In vitro treatment against 4T1 cells. **A** Relative cell activity after dealing with raised concentration of CaO_2_@mPDA-SH or mPDA nanoparticles. **B** Time-dependent confocal photos of 4T1 cells treated with CaO_2_@mPDA-SH (100 µg mL^−1^) or mPDA (100 μg mL^−1^) nanoparticles. Scale bar: 100 μm. **C** Time-dependent cellular uptake of 4T1 cells treated with CaO_2_@mPDA-SH (100 μg mL^−1^) nanoparticles examined by flow cytometry. **D** Apoptosis ratios of flow cytometry apoptosis experiment based on annexin V-FITC/PI staining of 4T1 cells after incubation with different concentration of CaO_2_@mPDA-SH or mPDA nanoparticles. **E** CLSM images of 4T1 cells with hypoxia detection probes [Ru(dpp)_3_]Cl_2_ in different treatments. Scale bar: 50 μm. **F** Quantitative analysis of mean fluorescent intensity of treated cells of **E** (n = 5, mean ± SD). **G** Lactic acid consumption effect of mPDA, Ca(OH)_2_@mPDA-SH, CaO_2_@mPDA, and CaO_2_@mPDA-SH nanoparticles at cellular level

To determine the dosage at which the nanoparticles would exert its optimal therapeutic effect in vitro without inducing cell death directly, murine breast cancer cell line 4T1 was selected to detect the cytotoxicity of CaO_2_@mPDA-SH by CCK-8 firstly. 4T1 cells were cocultured with varying concentrations of mPDA and CaO_2_@mPDA-SH, respectively. As shown in Fig. 2A, mPDA exhibited good biocompatibility. Even if the mPDA concentration reached 400 μg/mL, almost no cytotoxicity appeared. Low concentrations of CaO_2_@mPDA-SH show little toxicity to cells, while the cell viability of the CaO_2_@mPDA-SH group was 61.7% when the concentration was enhanced to 200 μg/mL, which was mainly due to the higher CaO_2_ payloaded in cells resulted in overloaded calcium stress leading to cell death [27]. Apoptosis was detected by annexin V-FITC/PI staining (Fig. 2D, Additional file 1: Fig. S6). When the concentration of the CaO_2_@mPDA-SH group reached 200 μg/mL, it showed significant cell apoptosis with the apoptosis rate of about 27.41%, which was in accordance with the cytotoxicity results. Hence, CaO_2_@mPDA-SH at a concentration of 100 μg/mL was used for the subsequent in-vitro experiments.

### Cellular uptake of CaO_2_@mPDA-SH

In order to evaluate whether CaO_2_@mPDA-SH can enter the cells consume intracellular lactic acid, the cellular uptake of Rhodamine B-labeled CaO_2_@mPDA-SH was examined by fluorescence microscopy and flow cytometry (Fig. 2B, C, Additional file 1: Fig. S7). The results showed that both groups exhibited remarkably intracellular fluorescence intensity (FI) over time, while the difference is that mPDA reaches its relatively high FI value in a relatively rapid manner within 1 h mainly due to the outstanding wet adhesion and biocompatibility properties of mPDA [18], which is benefit for a faster uptake by tumor cell, while CaO_2_@mPDA-SH reaches its maximum value at 4 h in 4T1 murine cells. Meanwhile, phagocytic behavior shown that in the presence of micropinocytosis inhibitor (cytochalasin D, 10 × 10^–6^ M), the uptake efficiency dropped (Additional file 1: Fig. S8), which indicates that the CaO_2_@mPDA-SH nanoparticles could be phagocytosed via the micropinocytosis mediated pathway and have the potential to enter cells for intracellular lactic acid consumption.

### In vitro hypoxia improvement and lactic acid reduction

To further demonstrate the effect of CaO_2_@mPDA-SH on improving hypoxia, 4T1 cells were incubated with mPDA and CaO_2_@mPDA-SH. [Ru(dpp)_3_]Cl_2_, an intracellular O_2_ level indicator, was used to detect the changes in the intracellular environment. The red fluorescence of the indicator was quenched in the presence of oxygen [29, 30]. Confocal laser scanning microscope (CLSM) images showed that the cells treated by mPDA showed a strong red fluorescence intensity (Fig. 2E, F), indicating a highly hypoxic intracellular environment. In contrast, the cells treated with CaO_2_@mPDA-SH showed much lower red fluorescence, indicating that the degree of oxygen deficiency in the cells was significantly improved.

The lactic acid consumption effect of CaO_2_@mPDA-SH was evaluated at the cellular level. CaO_2_@mPDA without sodium hyaluronate modification, Ca(OH)_2_@mPDA-SH and mPDA nanoparticles were also evaluated as controls. As shown in Fig. 2G, it was observed that CaO_2_@mPDA and CaO_2_@mPDA-SH were more efficient than Ca(OH)_2_@mPDA-SH or mPDA in consuming lactic acid. In particular, when the concentration reached 100 μg/mL, the groups that contained CaO_2_ consumed about 90% of the lactic acid produced by the cells. The above results demonstrated that the CaO_2_@mPDA-SH had a promising ability to deplete the lactic acid and amended hypoxia in vitro.

### Antitumor effect of CaO_2_@mPDA-SH

**Fig. 3:**
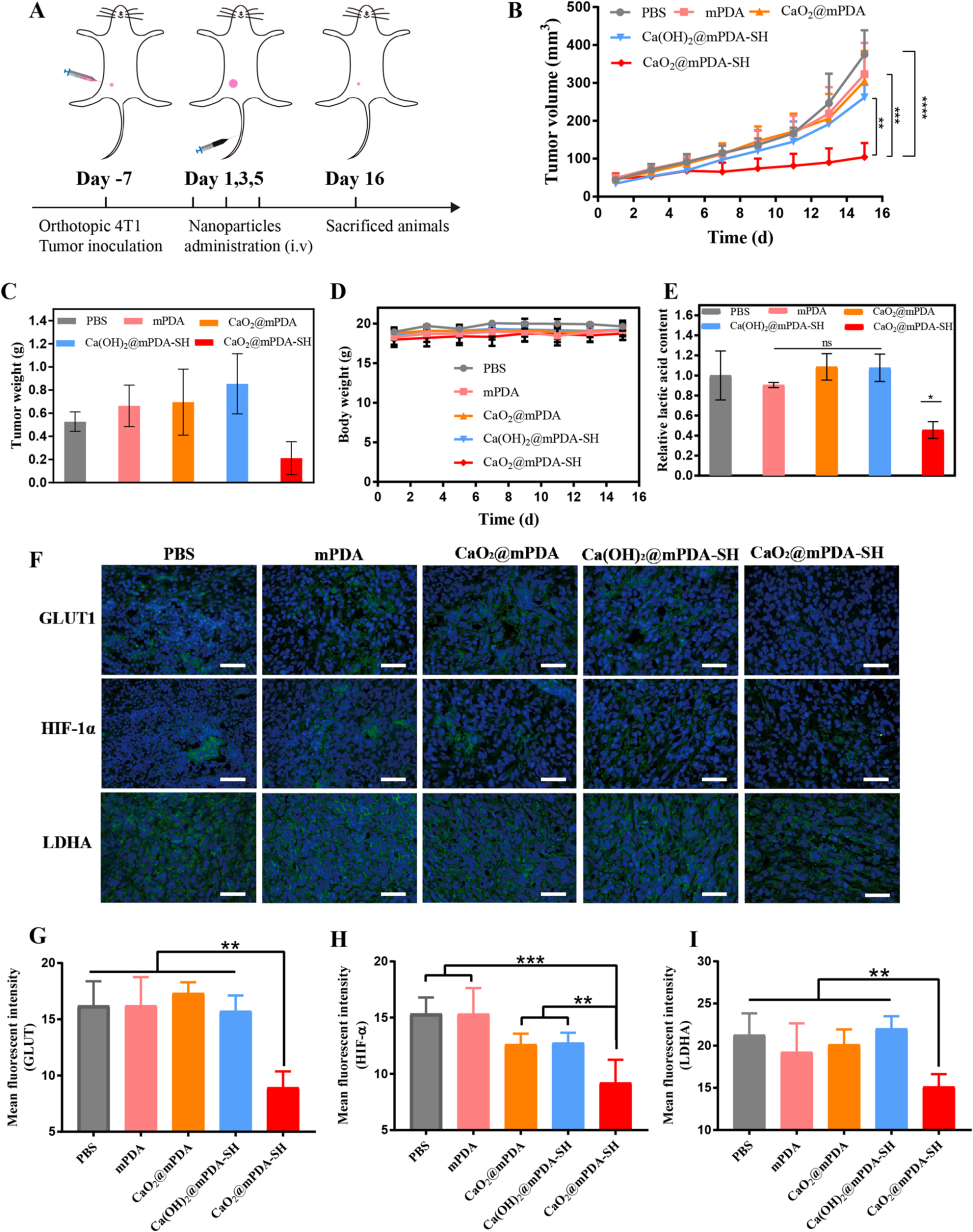
Antitumor effect study in 4T1 tumor models. **A** Diagrammatic representation of the therapeutic experiment (n = 5, mean ± SD). Tumor inoculation and intravenous injections were performed as described. **B** Comparison of tumor growth profiles in different treatments. **C** Average tumor weight after various treatment. **D** Mice weight changes during different treatments. **E** Tumor lactic acid concentration after different treatments. **F** Immunofluorescence staining of GLUT1, HIF-1α, and LDHA, respectively (Green: antibody, Blue: DAPI, Scale bar: 50 μm). **G**–**I** The corresponding quantitative expression analyses of **F**

We further studied the antitumor effect of CaO_2_@mPDA-SH in vivo. Mice were randomly divided into five groups. When the tumors reached 50 cm^3^, the nanoparticles were intravenously administered on the 1st, 3rd, 5th day, respectively (Fig. 3A). Changes in tumor volume and weight of mice were recorded (Fig. 3B, D). The tumor volume of the CaO_2_@mPDA-SH group was much smaller than that of the other four groups. On day 15, the tumors were removed and weighed (Fig. 3C). The therapeutic effects of mPDA group could be neglected, while the CaO_2_@mPDA group manifested undesirable anticipated effects, which is likely due to that without coating by sodium hyaluronate, CaO_2_ leaks out before reaching the tumor site. The growth of tumors was partly inhibited in the Ca(OH)_2_@mPDA-SH group for the inhibition rate was 30.23% through calculation (Additional file 1: Fig. S12). This indicates that consumption of lactic acid only without ameliorating hypoxia has limited inhibitory effect on tumor growth. The inhibition efficiency of tumors growth in the CaO_2_@mPDA-SH group was significantly improved with a tumor inhibition rate of 72.2% due to the intratumoral consumption of lactic acid and hypoxia amelioration. In in vivo biodistribution study, results showed that CaO_2_@mPDA-SH NPs could access to tumor via EPR effect (Additional file 1: Fig. S9) and exhibited long circulation time in the female Balb/c (Additional file 1: Fig. S10).

The underlying therapeutic mechanism of CaO_2_@mPDA-SH could rely on the reaction of CaO_2_ with lactic acid at the tumor site, which consumes the lactic acid and produces oxygen to attenuate hypoxia at the tumor site. Therefore, after different treatments, the tumor lactate concentration was firstly tested. As shown in Fig. 3E, a relatively lower lactate level in tumor was detected in the CaO_2_@mPDA-SH treatment group compared to the other control groups, roughly half the content of PBS group. It has been reported that HIF-1α contributes to lactic acid production by up-regulating the expression of GLUT1 and LDHA [31, 32]. To further explore the therapeutic mechanism of CaO_2_@mPDA-SH, we evaluated the expression of HIF-1α as well as related GLUT1 and LDHA by immunofluorescence staining. As shown in Fig. 3F, H, high HIF-1α expression was observed in control groups, while less expression was displayed in CaO_2_@mPDA-SH treated group. Accordingly, GLUT1 and LDHA also revealed relatively lower expression in CaO_2_@mPDA-SH treated group compared to other groups (Fig. 3G, I). The above evidences clearly proved that decreased lactate and hypoxia level in the tumor microenvironment leads to significant inhibition of tumor growth by the CaO_2_@mPDA-SH treatment group.

Finally, the biosafety was preliminarily assessed in vivo (Figs. 3D and Additional file 1: Fig. S13). There was no signal difference in body weight among the groups and no body weight loss caused by systemic toxicity. The tissues of the heart, liver, spleen, lung, and kidney were examined by H&E staining. There were no apparent inflammations and organ damages in the treatment groups compared with the PBS group.

### Activation of tumor immune microenvironment

**Fig. 4:**
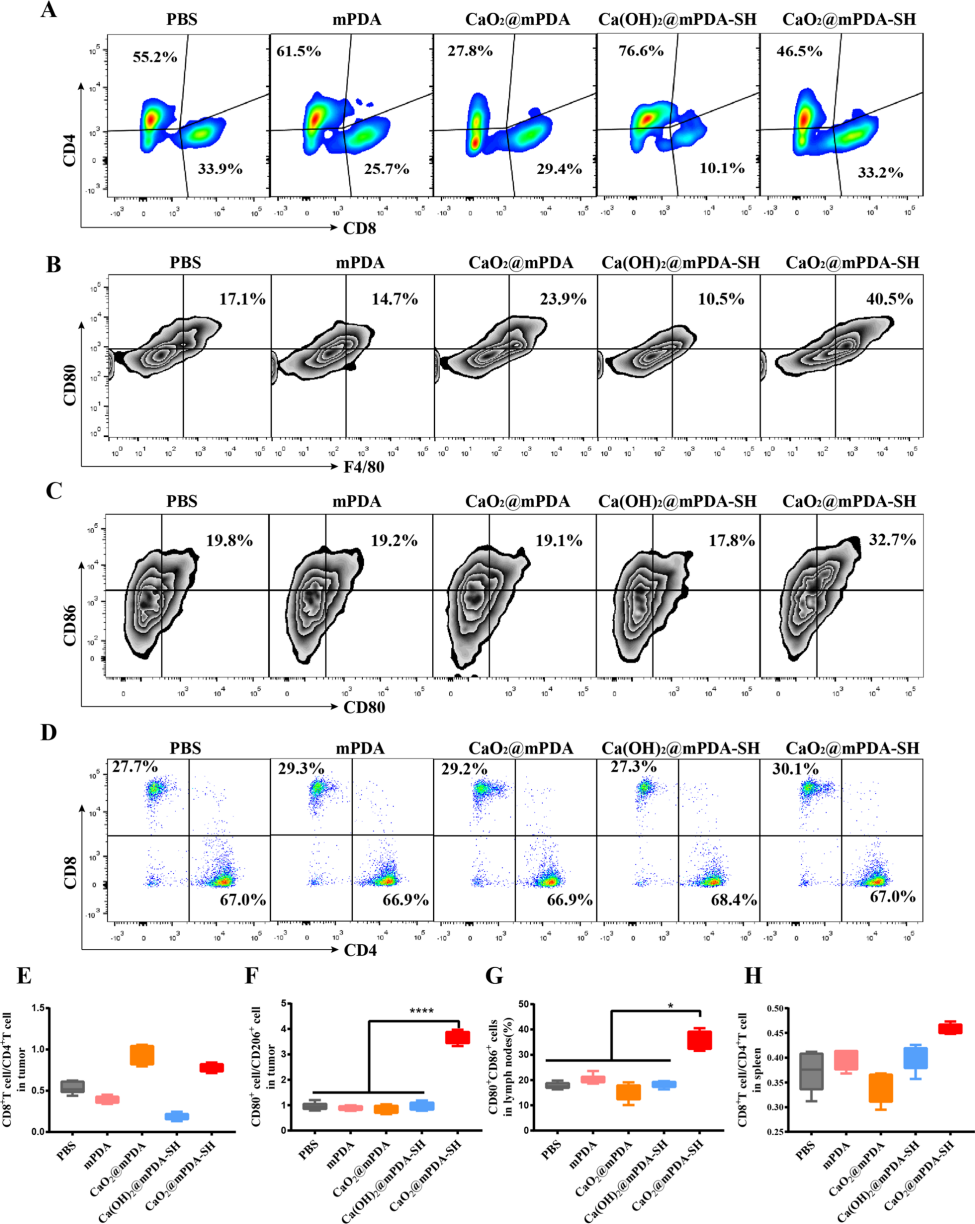
Immune analyses. **A** Representative FACS plots of tumor infiltration T cells (gated by CD3). **B** Representative FACS plots of tumor infiltration M1 macrophages (gated by CD11b). **C** Representative FACS plots of mature DCs in tumor-draining lymph nodes (gated by CD11c). **D** Representative FACS plots of T lymphocytes in the spleen (gated by CD3). **E** The ratio between CD3^+^CD8^+^ and CD3^+^CD4^+^ T cells in tumor for different groups according to the FACS data (n = 5). **F** The ratio between M1 and M2 macrophages of different groups according to the FACS data (n = 5). **G** Quantitative analyses of the CD80^+^CD86^+^ T cells in tumor-draining lymph nodes (n = 5). **H** The ratio between CD3^+^CD8^+^ and CD3^+^CD4^+^ T cells in spleen for different groups according to the FACS data (n = 5). In **E**–**H**, statistical significance was calculated through the one-way ANOVA with Tukey post-hoc analysis, *p < 0.05, **p < 0.01, ***p < 0.001

Subsequently, the immune responses after different treatments in tumor tissues were evaluated. To this end, the typical cell types of cellular immunity (T cells) and innate immunity (macrophages) were detected by flow cytometry. As shown in Fig. 4A, E, CaO_2_@mPDA-SH induces the infiltration of T cells to the tumor site due to the intratumoral consumption of lactic acid and hypoxia amelioration, thus promoted a significant up-regulation of the CD8^+^/CD4^+^ ratio, which is related to the prognosis of a variety of cancers and the response to immunotherapy, indicating that the tumor is in an active immune state [33]. For tumor infiltration macrophages, despite the absence of significant difference in the amount of M2 macrophages (Additional file 1: Fig. S14), the proportion of M1 macrophages increased in CaO_2_@mPDA-SH groups (Fig. 4B). The ratio between M1 macrophages and M2 macrophages was further calculated. The highest ratios in CaO_2_@mPDA-SH groups also demonstrated that the nanoparticles could improve the immunosuppressive TME (Fig. 4F).

Besides, we explored whether there were more mature DCs in tumor-draining lymph nodes (DLNs) in vivo by flow cytometry. The results indicated that the proportion of CD86^+^CD80^+^ DCs in the CaO_2_@mPDA-SH group increased, significantly enhanced the maturity of DCs compared with other control groups (Fig. 4C, G). Since spleen is the largest immune organ and the immune center of the body, we investigated the number and phenotype of T lymphocytes in the spleen subsequently. According to the quantitative analysis, CaO_2_@mPDA-SH significantly increased the ratio of CD8^+^T/CD4^+^T (Fig. 4D, H), suggesting that stimulation the systemic antitumor immunity. Results presented above show that CaO_2_@mPDA-SH could stimulate the maturation of DCs and induce the infiltration of CTLs into the tumor, thus exerting antitumor immune responses.

### Anti-metastatic and anti-angiogenesis effect

**Fig. 5:**
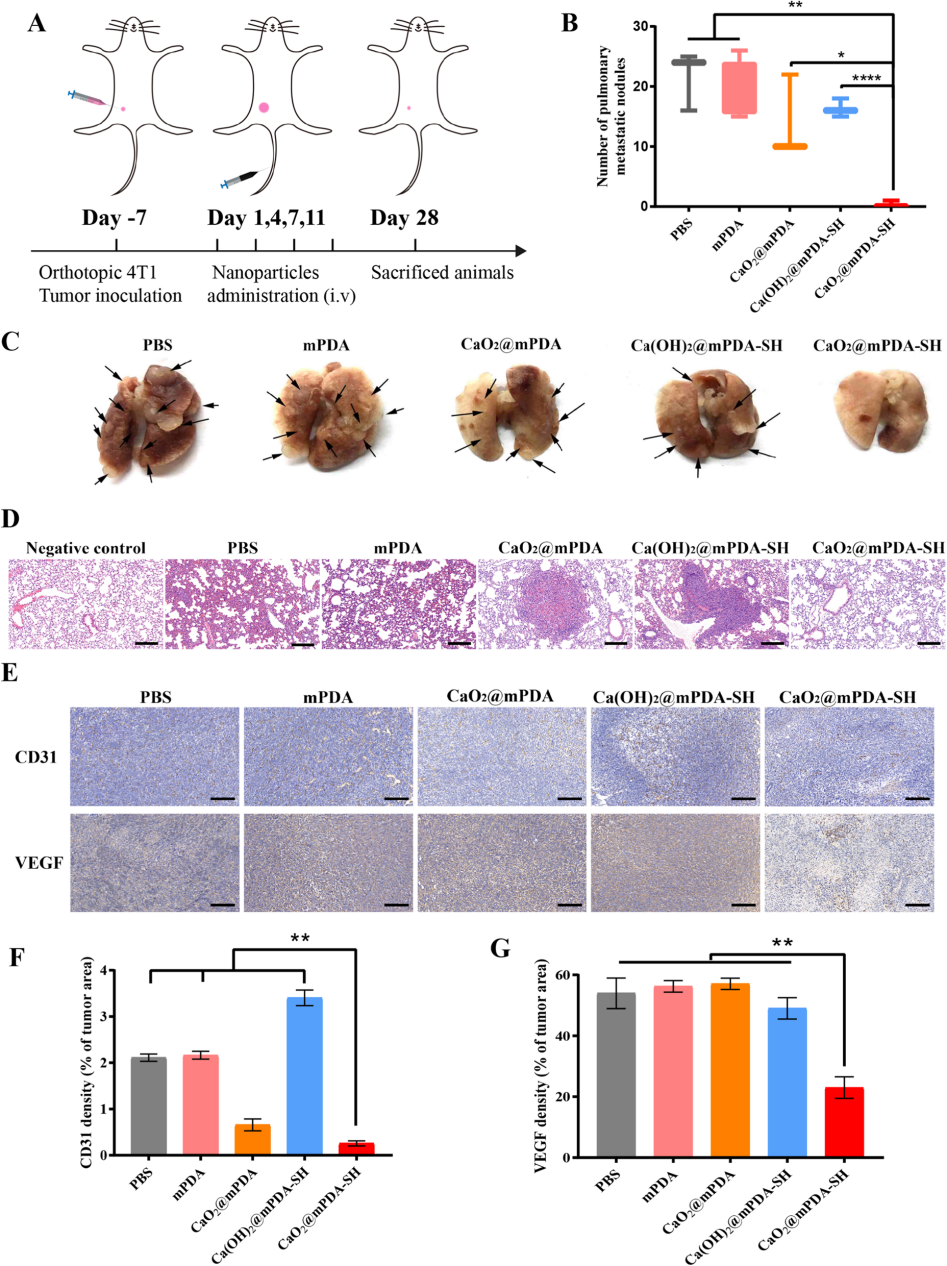
Anti-metastatic and anti-angiogenesis effect. **A** Diagrammatic representation of the therapeutic experiment (n = 5, mean ± SD). Tumor inoculation and intravenous injections were performed as described. **B** Number of lung metastases tumor nodules from different treatment groups. **C** Representative digital images showing the formation of tumor nodules in the lungs. Arrowheads indicate macroscopically detected pulmonary metastasis foci. **D** H&E staining of lung sections from different treatment groups. Scale bar: 200 μm. **E** Immunohistochemistry staining displayed expression of CD31 and VEGF in tumor tissues after different treatments. (Brown: antibody, Blue: cell nucleus, Scale bar: 200 μm). **F** Numbers of blood vessels/field and **G** percentages of VEGF stained area were analyzed in tumor sections. Results are the mean ± SD of 5 experiments. *P < 0.05, **P < 0.01, ***P < 0.005, ****P < 0.001

In addition to the antitumor efficacy, the lactate depletion mediated anti-metastatic and anti-angiogenesis effect of CaO_2_@mPDA-SH was also evaluated. When the tumors reached 50 mm^3^, such nanoparticles were intravenously administered on the 1st, 4th, 7th, 11th day, respectively (Fig. 5A). On day 28, the lungs were removed to photograph and count pulmonary metastatic nodules (Fig. 5B, C). It is noted that, the average number of pulmonary metastatic nodules in the PBS group was about 25, while no visible tumors nodules were observed the lungs of mice in CaO_2_@mPDA-SH group, indicating that the nanoparticles could significantly decrease the numbers of metastatic nodules in the lungs of treated mice compared with the PBS group. Furthermore, metastatic nodules were observed in the H&E micrographs of the lung in groups treated with PBS (Fig. 5D). Compared to the normal mice (negative control), all mice in PBS and other control groups developed various degrees of pulmonary metastases, while the CaO_2_@mPDA-SH significantly reduced tumor nodules. These results clearly validate that the CaO_2_@mPDA-SH mediated lactate-depleting enabled the reinforcement of the anti-metastasis performance.

Angiogenesis plays a critical role in the development and progression of cancers for delivering nutrients and oxygen to tumor, as well as allowing tumor cells dissemination to distant organs [34]. Given the critical role of lactate in promoting tumor angiogenesis, an immunohistochemistry approach was adopted to determine whether lactate depletion could suppress angiogenesis in treated tumors. As shown in Fig. 5E, CD31 expression, a marker of endothelial cells on blood vessels, was highly expressed in the PBS control group, but markedly attenuated in CaO_2_@mPDA-SH treated 4T1 tumors. Moreover, the expression of positive spots of VEGF, the primary regulator of angiogenesis, was also significantly reduced in the CaO_2_@mPDA-SH group. Tumor angiogenesis was also analyzed by quantifying the numbers of blood vessels/field and percentages of VEGF stained area via ImageJ software. Signal density for CD31 (Fig. 5F) and VEGF (Fig. 5G) within the tumor tissues were significantly lower in CaO_2_@mPDA-SH treated mouse than that in PBS group, indicating that due to the improvement of the intratumoral hyperlactic and hypoxic microenvironment, CaO_2_@mPDA-SH could significantly inhibit tumor vascular proliferation as well as control tumor metastasis.

## Conclusion

In summary, we have developed calcium peroxide-loaded mesoporous polydopamine nanoparticles modified with sodium hyaluronate (CaO_2_@mPDA-SH). These nanoparticles exhibited significantly dual regulation capability to the tumor microenvironment, including lactic acid consumption and hypoxia amelioration. On the one hand, CaO_2_@mPDA-SH nanoparticles exert antitumor immune responses by stimulating the maturation of DCs and increasing the proportion of M1 macrophages, and thus resulting in tumor growth inhibition; On the other hand, CaO_2_@mPDA-SH exert its antiangiogenic and anti-metastatic activities through the downregulation of VEGF and CD31 expression. Moreover, the relief of hypoxia could further reduce lactic acid production from the source by down-regulating the expression of HIF-1α, which further down-regulated the expression of glycolysis-associated enzymes includes GLUT1 and LDHA. This strategy provides a new therapeutic approach for tumor treatment.

## Supplementary Information

**Additional file 1.** Photograph of reaction solution before and after synthesis; hemolysis photograph mPDA and CaO_2_@mPDA-SH samples; hemolysis ratio; flow cytometry apoptosis experiment of 4T1 cells after incubation with mPDA and CaO_2_@mPDA-SH; cellular uptake of 4T1 cells treated with mPDA nanoparticles examined by flow cytometry; tumor inhibition ratio; H&E images of main organ sections; flow cytometric analysis of M2 macrophages in the tumor.

## Data Availability

The datasets used and/or analyzed during the current study are available from the corresponding author on reasonable request.
