# Correction: Acidic and hypoxic tumor microenvironment regulation by CaO_2_-loaded polydopamine nanoparticles

**DOI:** 10.1186/s12951-023-01934-y

**Published:** 2023-06-07

**Authors:** Shuangrong Ruan, Weimin Yin, Jiao Chang, Yan Yang, Jiuyuan Sun, Xiaoyi Ma, Ying Liu, Jie Zang, Yiqiong Liu, Yongyong Li, Tianbin Ren, Haiqing Dong

**Affiliations:** 1 Key Laboratory of Spine and Spinal Cord Injury Repair and Regeneration, Ministry of Education, School of Medicine, Tongji Hospital, The Institute for Biomedical Engineering & Nano Science, Tongji University, 389 Xincun Road, Shanghai, 200092 China; 2 School of Medicine, Shanghai Skin Disease Hospital, Tongji University, Shanghai, 200092 China

**Correction: Journal of Nanobiotechnology (2022) 20:544** 10.1186/s12951-022-01752-8

Following publication of the original article [1], the authors identified an error in affiliation. The revised affiliations are provided below.

1. Key Laboratory of Spine and Spinal Cord Injury Repair and Regeneration, Ministry of Education, School of Medicine, Tongji Hospital, The Institute for Biomedical Engineering & Nano Science, Tongji University, 389 Xincun Road, Shanghai 200092, China
2. Shanghai Skin Disease Hospital, School of Medicine, Tongji University, Shanghai, 200092, China

The original article [1] has been updated.
