# Supplementary material for: Acidic and hypoxic tumor microenvironment regulation by CaO2-loaded polydopamine nanoparticles
Source: J Nanobiotechnology. 2022 Dec 28;20:544. doi: 10.1186/s12951-022-01752-8 (PMC9798656; doi:10.1186/s12951-022-01752-8)
Supplement: Supplementary file 1 — Additional file 1. Photograph of reaction solution before and after synthesis; hemolysis photograph mPDA and CaO2@mPDA-SH samples; hemolysis ratio; flow cytometry apoptosis experiment of 4T1 cells after incubation with mPDA and CaO2@mPDA-SH; cellular uptake of 4T1 cells treated with mPDA nanoparticles examined by flow cytometry; tumor inhibition ratio; H&E images of main organ sections; flow cytometric analysis of M2 macrophages in the tumor. [file 12951_2022_1752_MOESM1_ESM.doc]

**Additional file 1**

Acidic and hypoxic tumor microenvironment regulation by CaO_2_-loaded polydopamine nanoparticles

Shuangrong Ruan^a,#^, Weimin Yin^b,#^, Jiao Chang^a^, Yan Yang^b^, Jiuyuan Sun^a^, Xiaoyi Ma^a^, Ying Liu^b^, Jie Zang^a^, Yiqiong Liu^a^, Yongyong Li^a^,*, Tianbin Ren^a^,*, Haiqing Dong^b^,*

1. Key Laboratory of Spine and Spinal Cord Injury Repair and Regeneration, Ministry of Education, School of Medicine, Tongji Hospital, The Institute for Biomedical Engineering & Nano Science, Tongji University, 389 Xincun Road, Shanghai 200092, China
2. Shanghai Skin Disease Hospital, School of Medicine, Tongji University, Shanghai, 200092, China

# Equal contribution

**The correspondence author:**

Haiqing Dong, Ph.D Professor

The Institute for Biomedical Engineering & Nano Science (iNANO)

Tongji University School of Medicine, Shanghai, P.R. China

Email: inano_donghq@tongji.edu.cn


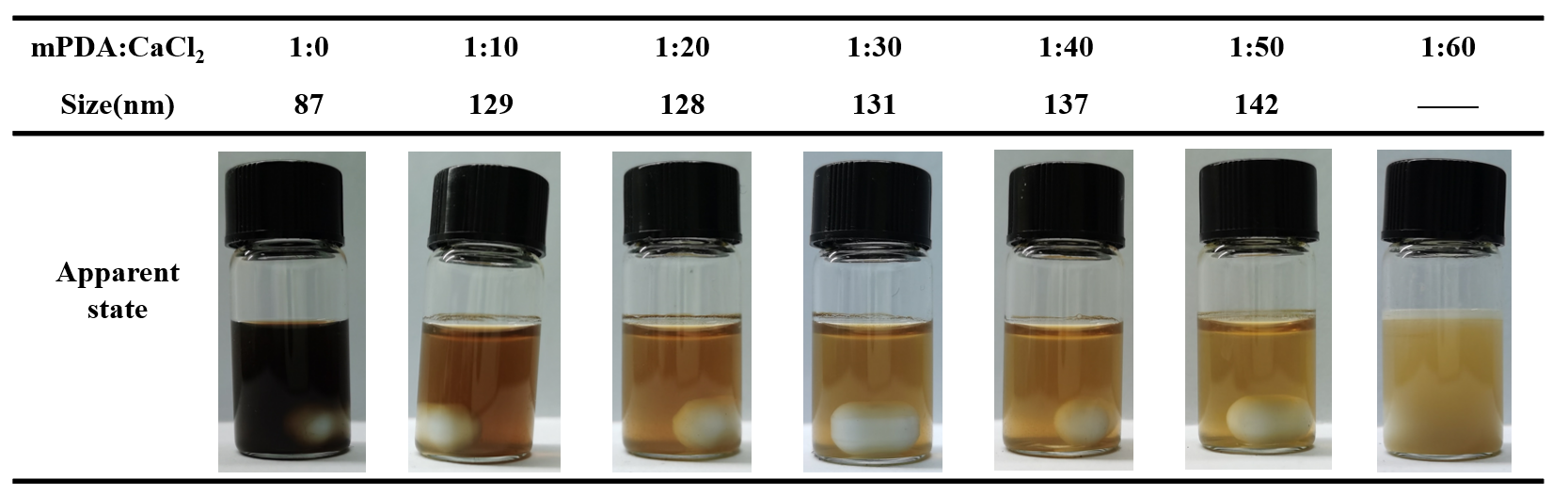


**Figure S1.** Color change and particle size after reaction with different mPDA/CaO_2_ feeding mass ratio.


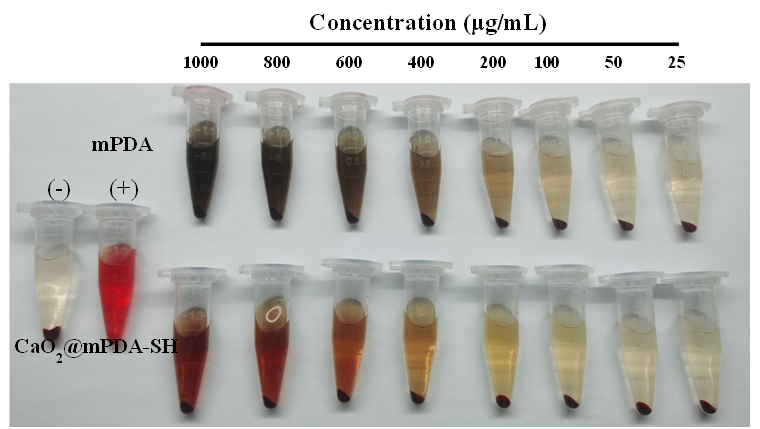


**Figure S2.** Hemolysis photograph at different concentrations of mPDA and CaO_2_@mPDA-SH samples.


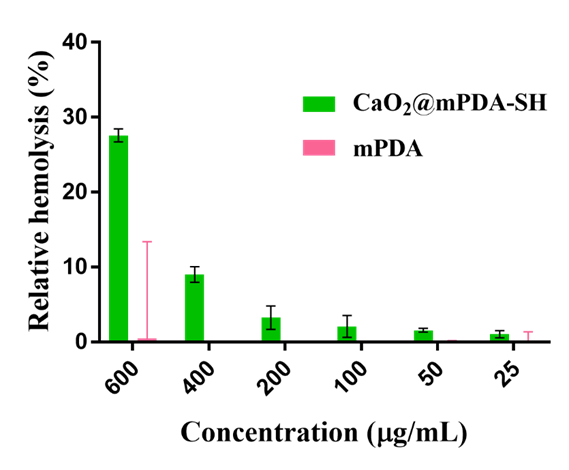


**Figure S3.** Hemolysis ratio at different concentrations. Results were expressed as mean ± SD.


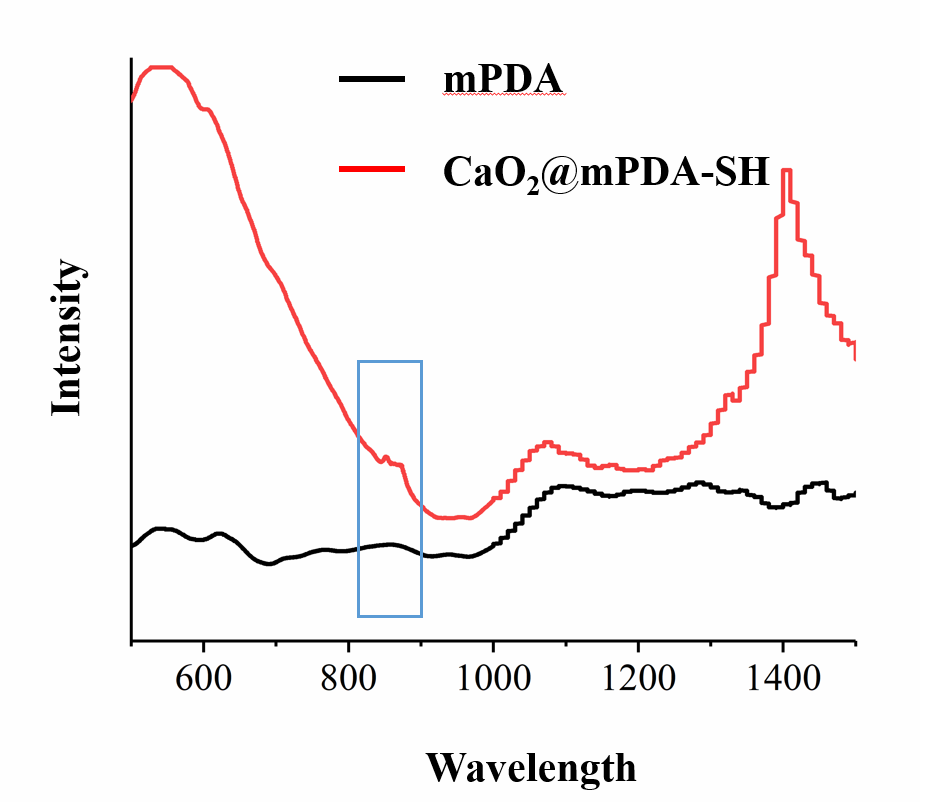


**Figure S4.** FTIR spectra of the mPDA and CaO_2_@mPDA-SH nanoparticles.

**
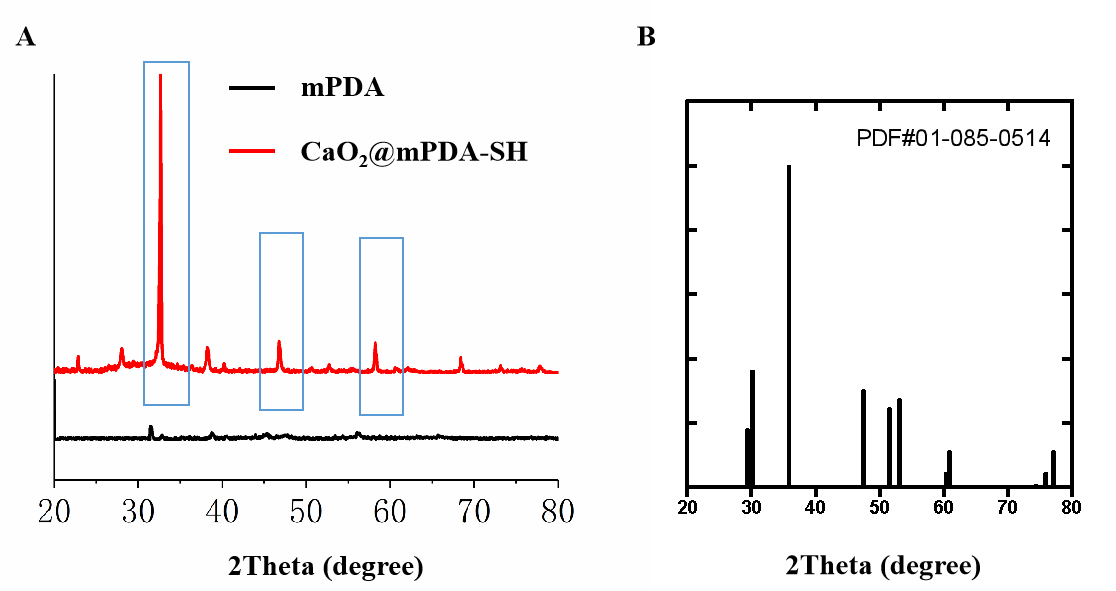
**

**Figure S5.** X-ray powder diffraction (XRD) pattern of A) the nanoparticles, B) the standard card of CaO_2_ (PDF#01-085-0514).


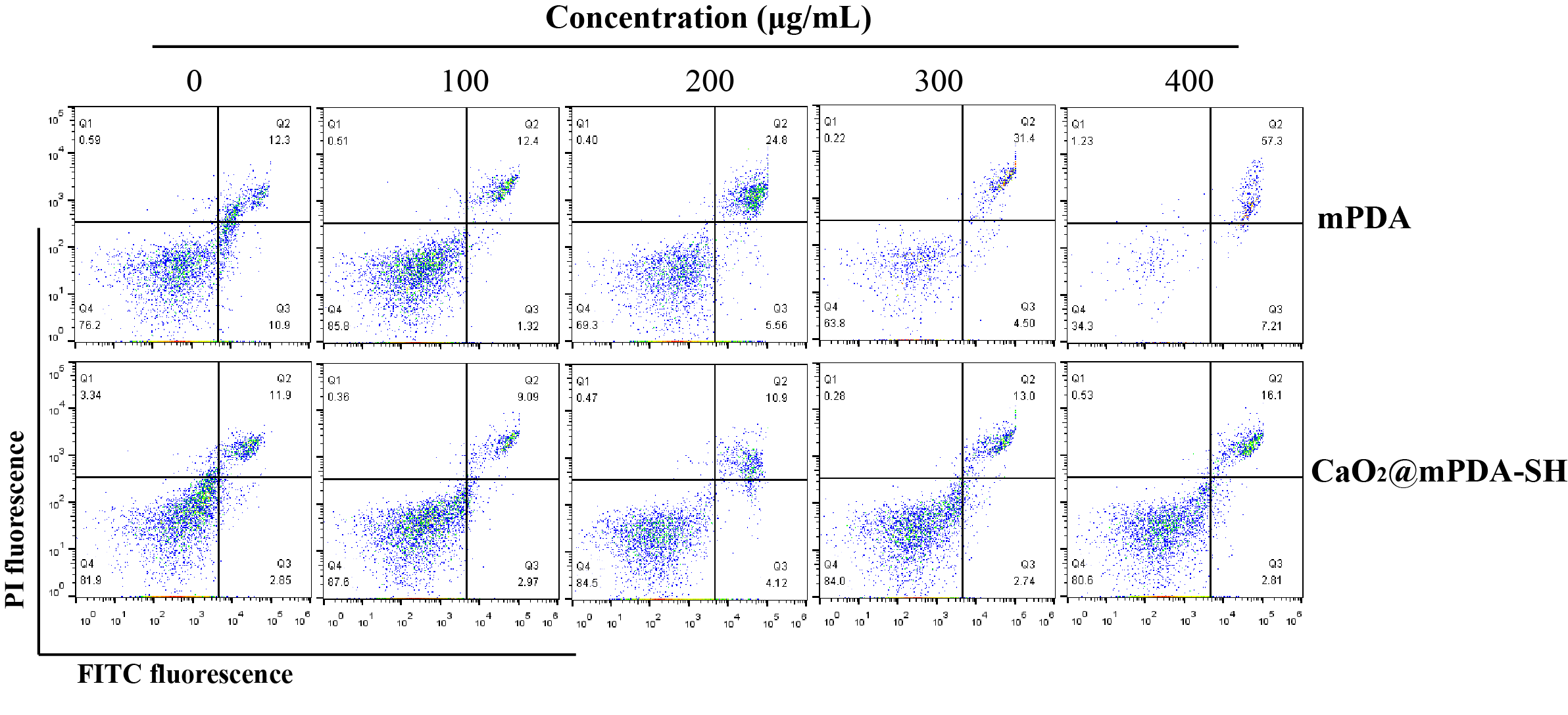


**Figure S6.** Flow cytometry apoptosis experiment based on annexin V-FITC/PI staining of 4T1 cells after incubation with different concentration.

**
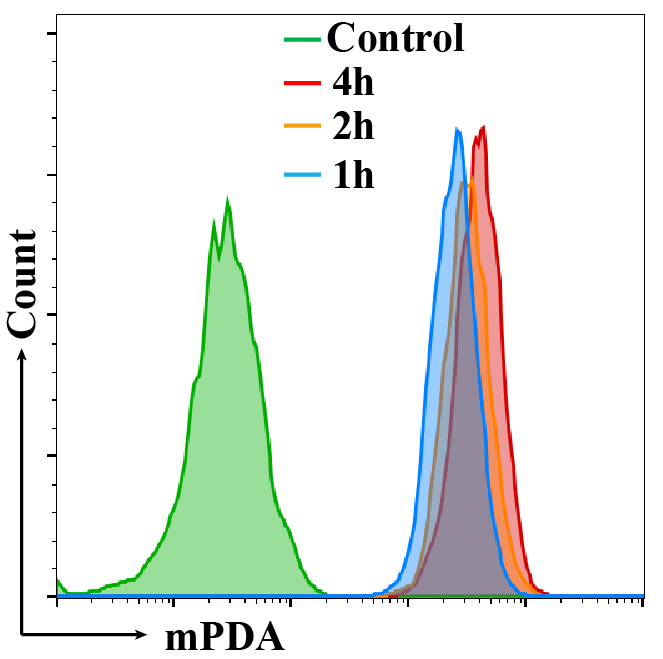
**

**Figure S7.** Time-dependent cellular uptake of 4T1 cells treated with mPDA (100 µg mL^−1^) nanoparticles examined by flow cytometry.

**
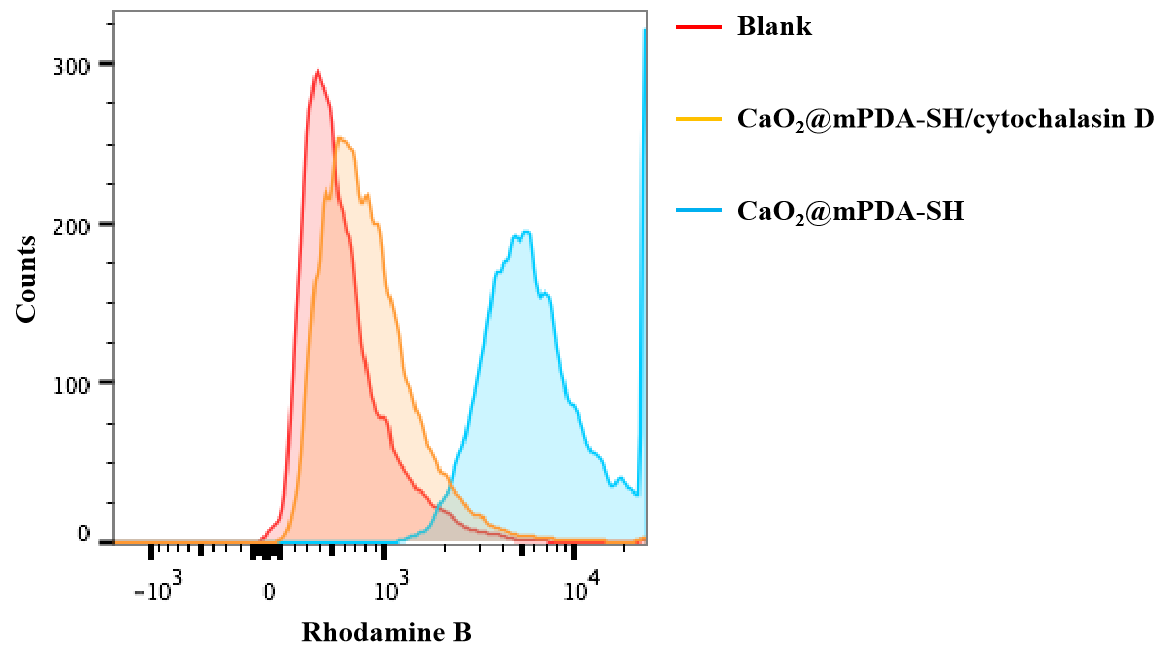
**

**Figure S8.** Macropinocytosis inhibitor (Cytochalasin D) decrease the uptake of the nanoparticles.

**
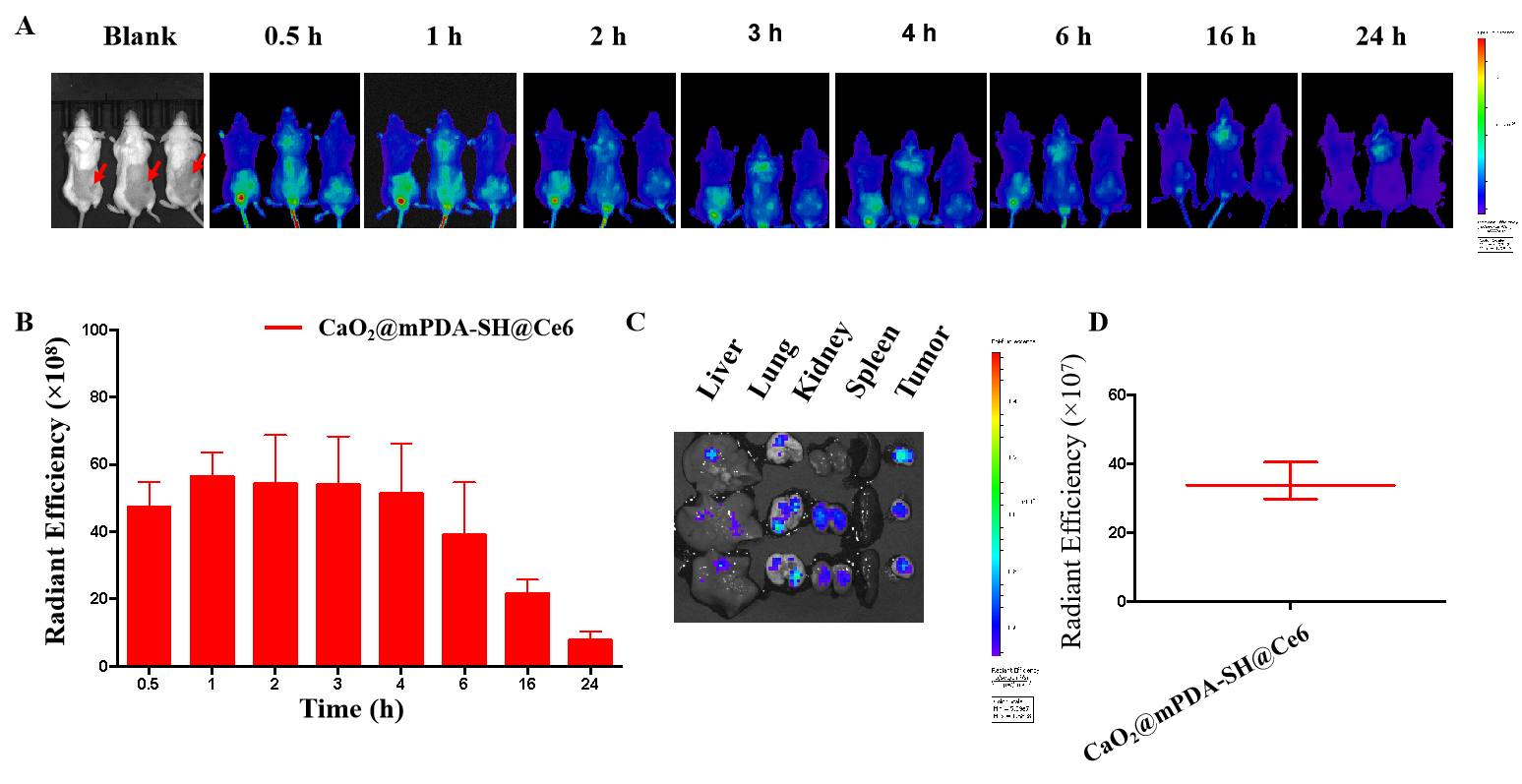
**

**Figure S9.** Biodistribution study. A) Living fluorescence imaging at different time intervals after i.v. injection of CaO_2_@mPDA-SH. B) In vivo radiant efficiency of tumor at different time intervals. C) Ex vivo imaging of major organs and the tumors (24 h). D) Ex vivo radiant efficiency of the tumors.

**
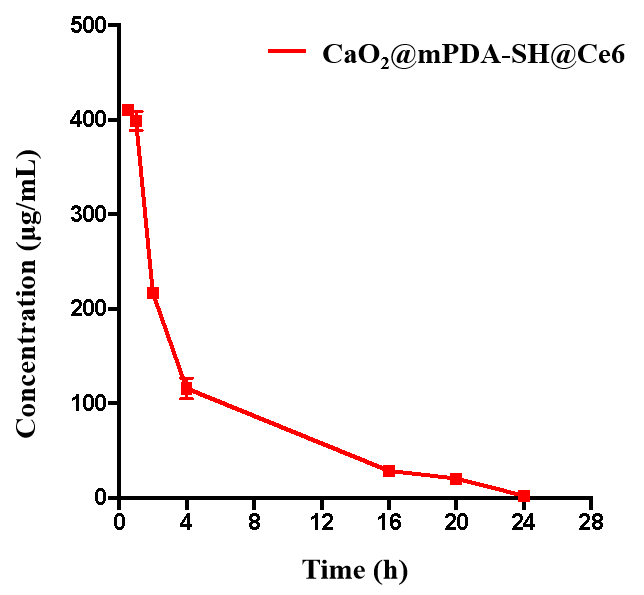
**

**Figure S10.** The concentration varition in blood of CaO_2_@mPDA-SH@Ce6 nanoparticles in the pharmacokinetics study.

**
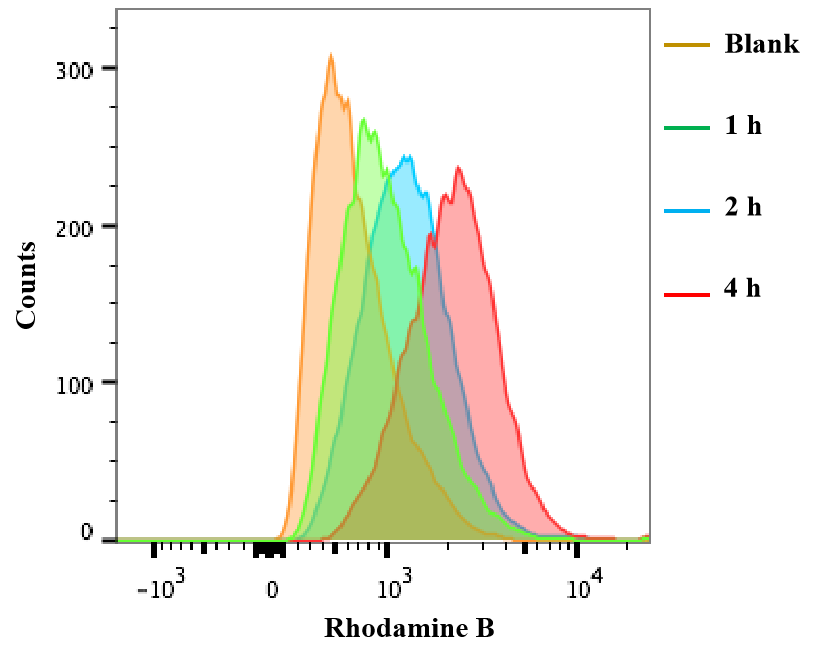
**

**Figure S11.** Time-dependent cellular uptake of human H1975 (NSCLC) cells treated with Rhodamine B labelled CaO_2_@mPDA-SH nanoparticles examined by flow cytometry.


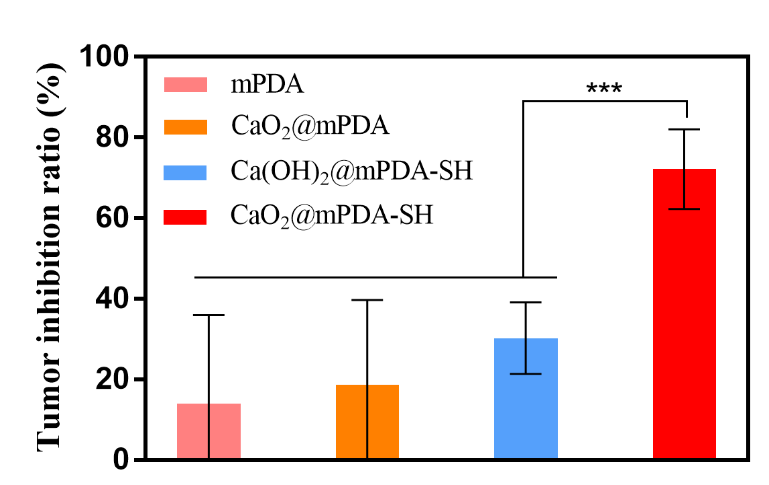


**Figure S12.** Tumor inhibition ratio in different groups.


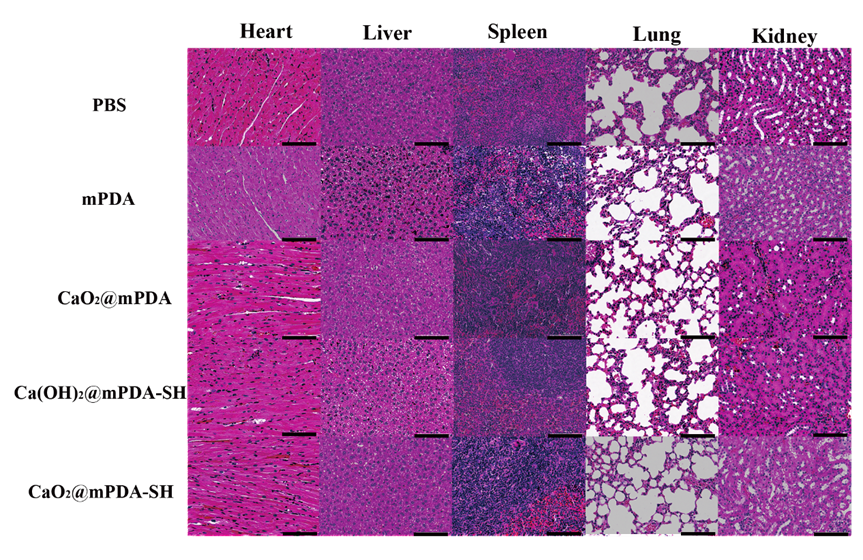


**Figure S13.** H&E staining of main organ sections at the end of treatment.


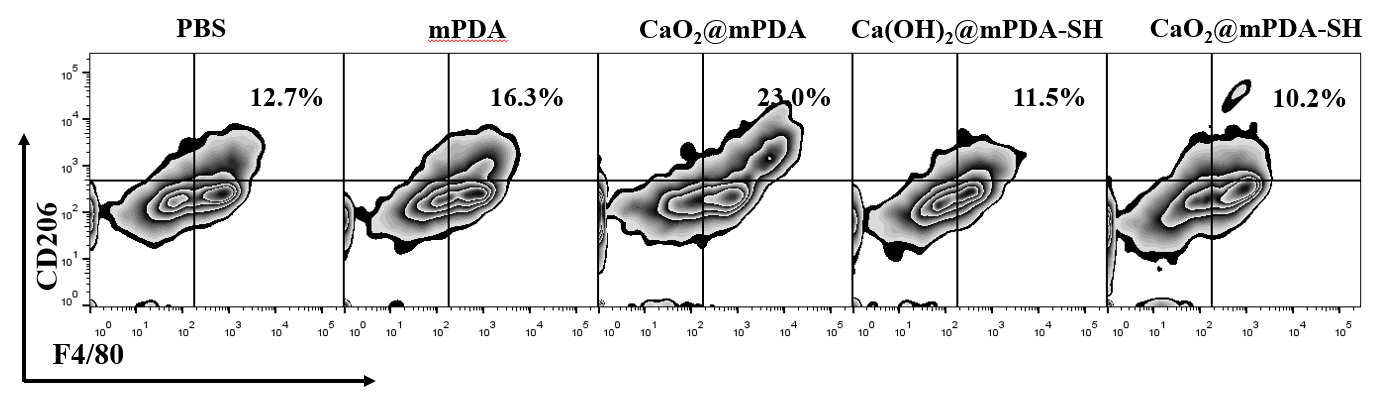


**Figure S14.** Representative FACS plots of tumor infiltration M2 macrophages (gated by CD11b)
